# Advanced deep learning framework for underwater object detection with multibeam forward-looking sonar

**DOI:** 10.1177/14759217241235637

**Published:** 2024-03-24

**Authors:** Liangfu Ge, Premjeet Singh, Ayan Sadhu

**Affiliations:** 1 Department of Civil and Environmental Engineering, The Western Academy for Advanced Research, Western University, London, ON, Canada; 2 Department of Civil and Environmental Engineering, Western University, London, ON, Canada

**Keywords:** Underwater infrastructure inspection, underwater object detection, multibeam forward-looking sonar imaging, deep learning, transfer learning, remotely operated vehicle, structural health monitoring

## Abstract

Underwater object detection (UOD) is an essential activity in maintaining and monitoring underwater infrastructure, playing an important role in their efficient and low-risk asset management. In underwater environments, sonar, recognized for overcoming the limitations of optical imaging in low-light and turbid conditions, has increasingly gained popularity for UOD. However, due to the low resolution and limited foreground-background contrast in sonar images, existing sonar-based object detection algorithms still face challenges regarding precision and transferability. To solve these challenges, this article proposes an advanced deep learning framework for UOD that uses the data from multibeam forward-looking sonar. The framework is adapted from the network architecture of YOLOv7, one of the state-of-the-art vision-based object detection algorithms, by incorporating unique optimizations in three key aspects: data preprocessing, feature fusion, and loss functions. These improvements are extensively tested on a dedicated public dataset, showing superior object classification performance compared to the selected existing sonar-based methods. Through experiments conducted on an underwater remotely operated vehicle, the proposed framework validates significant enhancements in target classification, localization, and transfer learning capabilities. Since the engineering structures have similar geometric shapes to the objects tested in this study, the proposed framework presents potential applicability to underwater structural inspection and monitoring, and autonomous asset management.

## Introduction

With growing demands of transportation and trade, underwater structures, such as undersea tunnels and bridges spanning oceans, have become an integral component of modern infrastructure. In addition, the demand for power generation accelerates the development of underwater infrastructure such as hydroelectric dams, offshore mining platforms, and underwater pipelines. Most of these structures are positioned in particularly challenging environments, in which their degrading process can be greatly accelerated by the adverse effects of wave scouring, biological activity, chemical corrosion, etc.^1^ Therefore, timely inspection and remote and automated structural health monitoring (SHM) are crucial to ensure the safety and longevity of the underwater infrastructure.^2–7^ In this article, an advanced deep learning framework is developed to undertake underwater object detection (UOD) using sonar images of a remotely operated underwater drone.

Compared with surface structures, inspecting underwater infrastructure faces significant challenges. Most of the underwater infrastructure assessments are currently performed by professional divers.^8^ Therefore, manual inspections are not only costly and time-consuming but also pose a potential safety risk to the inspectors. In addition, human divers can only reach depths of up to 100 m, making it challenging to monitor many deepwater structures.^9^ Moreover, factors such as strong currents, limited visibility, and adverse weather conditions further restrict the range of structures humans can inspect.^10^ To overcome these challenges, underwater unmanned vehicles (UUVs), such as remotely operated vehicles (ROVs) and autonomous underwater vehicles (AUVs), have been developed in recent years as an alternative for diver-based inspections.^11,12^ UUVs are subsea drones that incorporate various sensors and navigation systems, which can be applied to nondestructive testing and SHM. For specific applications in SHM, Ciszewski et al.^13^ proposed a modular, reconfigurable mobile robotic system for offshore construction monitoring, which is composed of two underwater robotic platforms, including a tracked robot for moving on rough terrain and an ROV for underwater operation. In this system, a 3D sonar was used for navigation, whereas optical cameras and laser sensors were applied for photographic data measurement. Xiang et al.^14^ developed an AUV navigation system with two tri-axial magnetometers to track subsea cables and presented a new analytic formulation to compute the heading deviation, horizontal offset and buried depth of the cable. The effectiveness of this system was validated through a numerical simulation. Jiao et al.^4^ proposed a real-time marine and offshore SHM system based on controllable underwater robots. A vision-based image processing module was integrated into the system to assess the damage to underwater concrete structures. As an important augmentation to traditional diver surveys, UUVs facilitate the exploration and inspection in deeper and more complex environments, rendering them well-suited for SHM of underwater infrastructure.

Object detection and recognition are central tasks in robotic underwater inspection, which play a crucial role in underwater navigation and damage identification. Optical imaging and sonar imaging are the two main types of data that UUV inspections are based on. Regarding optical imaging, Chen et al.^15^ proposed an object detection model using monocular vision sensors and suggested an extraction method for the regions of interest using light transmission information besides commonly used visual features such as color and intensity. Choi et al.^16^ developed an ROV by combining a high-definition optical camera with a new lighting system for harbor inspections. Its visual inspection performance was verified by experiments both in a basin and a sea trial. Huang et al.^17^ put forth an improved, faster region-based convolutional neural network and accurately detected concrete cracks, spalling, and precipitates by using optical images.

As monocular imaging cannot obtain depth information, some studies explored the application of 3D imaging. Drap et al.^18^ proposed a 3D modeling algorithm based on optical odometry and photos from three cameras. The method was validated in a full-scale study of an ancient shipwreck. In another vein, Hong et al.^19^ employed a stereo camera unit and an acoustic altimeter to create photomosaics and developed a visual inspection system for checking the structural integrity and biofouling of docked ships. Optical images provide intricate details in terms of color and texture, coupled with a high resolution, thereby offering a precise representation of the structural features. Furthermore, given the comprehensive research on computer vision techniques utilizing optical cameras across various SHM domains such as apparent detection,^20^ displacement measurement,^21^ and traffic load monitoring,^22^ optical-imaging-based object detection methodologies have evolved to become both mature and readily deployable. However, the quality and range of optical imaging are affected by the illumination and water turbidity (i.e., cloudiness in water), which can greatly limit the application of UUVs if only using camera sensing. To address these challenges, this article develops an objection detection methodology using the sonar images of UUV navigating in the underwater environment by leveraging the capabilities of deep learning.

Sonar imaging technology, which uses the reflection of underwater acoustic waves for object detection and distance measurement, can overcome the limitations inherent to optical imaging.^8^ In general, sonar-based object detection techniques can be divided into several categories: fathometers, sector scanning sonar, side-scan sonar, multibeam forward-looking sonar, etc.^23^ In the past decades, side-scan and forward-looking sonars were more commonly used, whereas some classic machine learning (ML) approaches were studied to solve object detection and segmentation in underwater environments. For instance, Song et al.^24^ introduced a novel method for segmenting side-scan sonar images, combining convolutional features with an extreme learning machine, a derivative of single-hidden layer feedforward neural networks. The method was demonstrated to outperform typical CNN and support vector machine (SVM). On the front of unsupervised ML approaches, Ye et al.^25^ used the Gauss–Markov random field model to extract local texture features in sonar images. Subsequently, they integrated local features into the level-set energy functions to segment shadow and highlight regions. Shi et al.^26^ put forth a detection and classification approach for underwater dam crack assessment using block clustering and statistical evaluations of sonar images. Although traditional ML techniques have proven effective in specific aquatic environments, their efficacy for comprehensive underwater inspections under intricate conditions remains constrained. Compared to optical images, sonar images that are created from the reflected waves are typically grayscale and have a lower resolution, resulting in the image features often appearing more indistinct and similar across various targets. Therefore, to ensure stable and precise object detection for real sonar images, it remains necessary to develop methods with better feature extraction capabilities.

Given their robust learning and generalization performance, deep learning techniques have been widely used in SHM.^27–31^ CNN-based approaches, as the most representative ones, have also garnered an increasing attention in UOD using sonars. Zhu et al.^32^ presented an automatic target recognition approach for UUVs equipped with sonar. In this approach, a CNN was used for feature extraction, followed by a trained SVM for the classification of targets. Neves et al.^33^ introduced a multi-object detection system that outputs object position and rotation from sonar images to support AUV navigation, combining YOLOv2 and the rotational attention mechanism. Yu et al.^34^ proposed a Transformer-YOLOv5-based model aiming to improve object detection robustness for side-scan sonar images. Regarding the specific applications, Xiong et al.^35^ employed a real-time 3D sonar system to conduct automatic monitoring, evaluation, and positioning of exposed subsea pipelines. They trained an object detector based on the YOLOv5 algorithm and localized the pipeline by using the spatial position mapping between the pipeline, the ROV, and the tracking ship. Meanwhile, Hou et al.^8^ developed a sonar-driven inspection framework for underwater bridge substructures, applying the U-Net architecture. They successfully validated that the framework could identify the scour depth and damages in a bridge foundation based on pixel-wise segmentation images. To support the comparative analysis of different object detection models, Xie et al.^36^ created an underwater acoustic target detection (UATD) dataset. The study also benchmarked Faster Region-based Convolutional Neural Network (RCNN) and YOLOv3 performance across various backbones using this dataset. With two similar sonar image datasets, Wang et al.^37^ proposed a multilevel feature fusion network and conducted extensive studies to validate its efficacy in multi-class object detection. It can be concluded from the above studies that incorporating multiscale features plays an important role in CNN-based sonar image object detection. These studies reported various models capable of image feature extraction at different scales and were validated using specific experiments or datasets. However, due to the influence of low resolution, shadows, and background noise of sonar images, the efficiency of these methods in feature fusion is not yet optimal, resulting in a significant gap in the precision of small object localization. On the other hand, since collecting sonar images for underwater structures is time-consuming and expensive, it is important to adopt the strategy of transfer learning when the CNN-based models are applied to unknown scenarios, reducing the training cost and improving their practicality in SHM applications. However, the transferability of CNN-based models to new datasets has rarely been explored in the existing studies of UOD.

Given the limitations of existing studies, this article introduces a novel deep-learning framework for UOD using forward-looking sonar. The framework is derived from the architecture of YOLOv7, a leading algorithm in computer vision.^38^ The proposed framework incorporates three enhancements aimed at bolstering multiscale feature fusion and improving the accuracy of small target localization. A series of ablation experiments are conducted on a public sonar image dataset named UATD to illustrate the superiority of the proposed approach over other state-of-the-art algorithms. Subsequently, this article presents the details of an experimental study on UOD using a sonar-equipped ROV, where the proposed deep learning framework is tested and validated on targets with similar shapes to underwater infrastructure. Notably, this marks the first instance where the transferability of the proposed approach is validated through the transfer learning between the UATD dataset and the experimental data.

The remainder of this article is organized as follows. Proposed methodology gives a brief introduction to the YOLOv7 algorithm and then details the improvements in the proposed framework. Model evaluation on the public dataset presents the ablation experiments to validate the advantages of the proposed approach. Experimental study introduces a UOD experiment using a ROV platform and the results of transfer learning of the proposed model. Finally, Conclusions concludes the highlights and contributions of this article.

## Proposed methodology

### YOLOv7

YOLOv7 is a one-stage object detection algorithm, which outperforms most renowned object detectors in both speed and accuracy in the range of 5–160 fps.^38^ Different from the other mainstream object detectors that are mostly developed based on architecture optimization,^39–41^ YOLOv7 is focused on some optimized modules and optimization methods, which may raise the training cost to improve the object detection accuracy, but without increasing the inference time. The architecture of YOLOv7 consists of three parts: Input, Backbone, and Head. In the Input part, some preprocessing operations such as mixup^42^ and mosaic^43^ are conducted for data augmentation, and then the images with united size are fed into the Backbone to extract features from three different scales. The image features extracted from different channels are then fused in the Head part and are finally used to predict the categories and positions of the targets in the input images.

**Figure 1.**
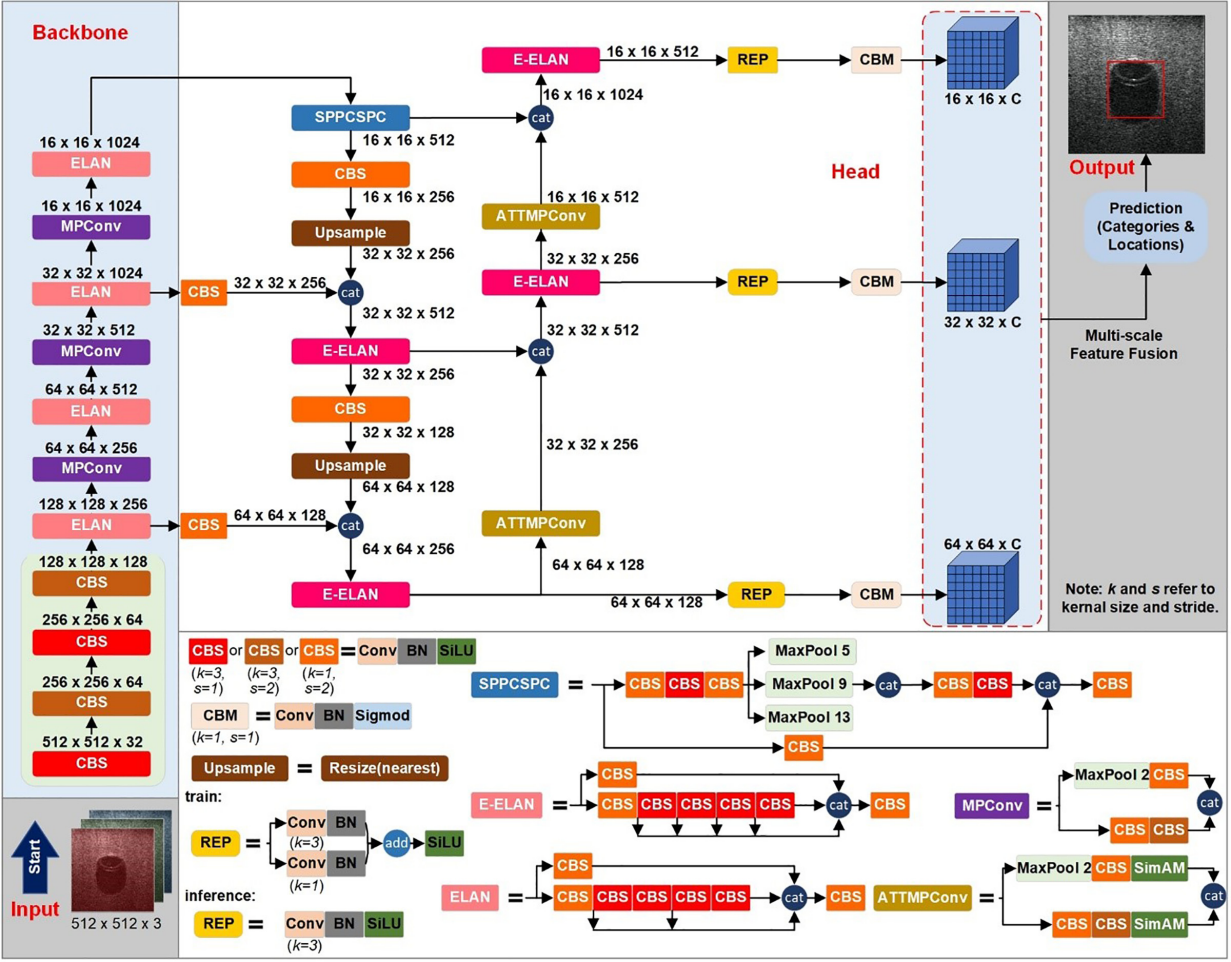
The proposed object detection framework.

There are several modules in the Backbone and Head of YOLOv7, including the CBS module, MPConv module, efficient layer aggregation networks (ELAN module), extended-ELAN (E-ELAN module), SPPCSPC module, and CBM module. Specifically, the CBS and CBM modules are combinations of a convolution layer, a batch normalization layer, and the activation functions SiLU^44^ and Sigmoid,^3^ respectively. The MPConv module combines a MaxPool layer and three CBS modules to form upper and lower branches. Two branches are subsequently merged using concat operation to enhance the feature extraction capability of the network. The SPPCSPC module integrates parallel MaxPool layers with a series of CBS modules. This module is used to avoid image distortion caused by down-sampling processing and to prevent extracting repeated features in CNNs. The ELAN module is used to optimize the gradient length of the overall network, while the E-ELAN module uses expand, shuffle, and merge cardinality to continuously enhance the learning ability of the network without destroying the original gradient path. The detailed architecture of these modules can be referred to in Figure 1.

The fundamental concept behind the YOLOv7 centers on enhancing the modules and refining the training process. This approach increases training costs to improve accuracy while maintaining the same inference time. However, the following points have not been fully considered in the original YOLOv7:

1. Anchors, which are predefined bounding box shapes with specific aspect ratios and sizes, play an important role in the YOLO series of target detectors. They enable the YOLO algorithm to adeptly manage the detection of objects with different sizes and shapes and provide a good starting point for model training. YOLOv7 follows the calculation method of anchors of the earlier versions of YOLO (i.e., the *k*-means algorithm). The quality of clustering with this approach is notably influenced by the selection of initial parameters.
2. YOLOv7 uses feature maps from three different scales for regression and prediction; however, it cannot perform adaptive learning on the contribution of multiscale features.
3. Limited by the loss function, the prediction accuracy for target locations still needs to be further improved, especially for small targets.

### The proposed UOD framework

Considering the limitations inherent in YOLOv7, this study introduces an enhanced object detection framework tailored for sonar imagery in complex underwater scenarios. The architecture and core modules are shown in Figure 1. The arrows and horizontal stacking of rectangles in the figure represent the forward propagation between modules.

In the proposed framework, the main body of YOLOv7 is retained. Within the input module, sonar images are resized into square shapes of 512 × 512 with three channels corresponding to RGB color space. As the model undergoes forward propagation, it ultimately produces three feature maps of dimensions 16 × 16 × *C*, 32 × 32 × *C*, and 64 × 64 × *C*, respectively. The channel of these feature maps *C* is given by

(1) C=(Numberofclasses+5)×3

In Equation (1), the number 5 indicates predicting five parameters, including four for bounding box offsets and one for objectness prediction. The number 3 represents that three bounding boxes are predicted at each scale. These feature maps are then used to calculate the value of the loss function, which includes three terms: classification loss $L_{\mathrm{cls}}$ , object detection confidence loss $L_{\mathrm{obj}}$ , and coordinate regression loss $L_{\mathrm{coord}}$ . The value of the loss function at three scales is fused according to the weight ratio of 0.4:1:4 as follows:

(2) $$L_{\mathrm{total}}=0.4L_{16\times 16}+L_{32\times 32}+4L_{64\times 64}$$

where $L_{s\times s}$ ( s=16,32,64 ) represents the loss calculated through feature maps in dimension s×s . *s* denotes the number of image pixels. Similarly, the three items in the loss function are combined according to different weights as below.

(3) $$L_{s\times s}=\lambda_{1}L_{\mathrm{cls}}+\lambda_{2}L_{\mathrm{obj}}+\lambda_{3}L_{\mathrm{coord}}$$

In Equation (3), $\lambda_{1}=0.5\times (\mathrm{Number}\;\mathrm{of}\;\mathrm{class}/80)$ , $\lambda_{2}=W\times H/640^{2}$ , $\lambda_{3}=0.05$ , where *W* and *H* denote the width and height of input images, respectively. The weight coefficients are selected based on the official recommendations of YOLOv7.^38^ To improve the learning ability for small targets and the prediction accuracy of target locations, the proposed model is enhanced from three aspects, that is, anchor calculation, multiscale feature fusion, and loss function, which are detailed in the following subsection.

### The proposed improvements for the original YOLOv7

#### Estimation of the anchors

In the typical training of YOLOv7, the *k*-means clustering algorithm is used to create three sets of anchors, corresponding to three different sizes of feature maps. The anchors provide a starting point for the regression of target locations. However, the *k*-means algorithm is inherently non-robust and sensitive to outliers, which may cause unstable clustering results and further influence the localization accuracy.^45^ Therefore, in the proposed model, the *k*-means++algorithm, which has been proven to outperform standard *k*-means in terms of both speed and accuracy, is explored. The steps of the *k*-means++ algorithm include^45^:

(a) Choose an initial center *c*_1_ uniformly at random from the set of data points X .
(b) Choose the next center *c_i_*, selecting $c_{i}=x^{,}\in X$ with probability $\frac{D{\left(x^{,}\right)}^{2}}{\sum_{x\in X}D{\left(x\right)}^{2}}$ . *D*(*x*) denotes the shortest distance from data point *x* to the closest center that has been chosen.
(c) Repeat Step (b) until all the *k* centers have been chosen.
(d) Perform the standard *k*-means algorithm.

The *k*-means++algorithm can greatly enhance the matching degree between the prior anchor boxes and the actual detection boxes, in turn improving the accuracy of localization.

#### Self-attention mechanism with a simple and parameter-free attention module

**Figure 2.**
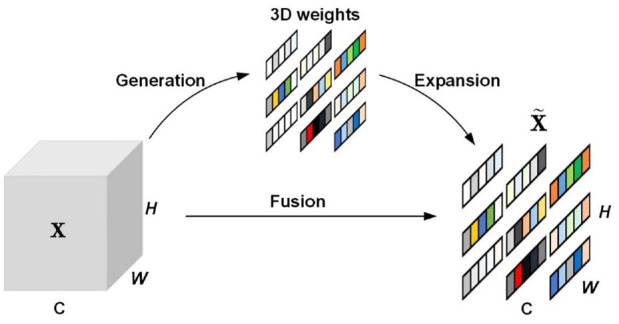
The attention steps of SimAM.

In the proposed framework, the ATTMPConv module is introduced to replace the original MPConv in YOLOv7, enhancing the feature-focusing capabilities of CNNs. As shown in Figure 1, the core of ATTMPConv lies in a simple and parameter-free attention module, which is named SimAM.^46^ In contrast to existing channel-wise and spatial-wise attention modules, SimAM can refine channel and spatial features simultaneously and infer 3D attention weights for feature maps without adding parameters to the original networks, as shown in Figure 2.

To implement attention, SimAM needs to estimate the importance of each neuron (i.e., each colored subregion plotted in Figure 2). In neuroscience, neurons exhibiting unique firing patterns distinct from their neighboring neurons are often regarded as the most informative, and an engaged neuron might also inhibit its surrounding neurons, a process termed spatial suppression.^47^ Based on these findings, the neurons showing spatial suppression should be assigned higher weights. To find out such neurons, the following energy function is defined for each neuron^46^:

(4) $$e_{t}\left(w_{t},b_{t},y,x_{i}\right)={\left(y_{t}-\hat{t}\right)}^{2}+\frac{1}{M-1}\sum_{i=1}^{M-1}{\left(y_{o}-{\hat{x}}_{i}\right)}^{2}$$

where, $\hat{t}=w_{t}t+b_{t}$ and ${\hat{x}}_{i}=w_{t}x_{i}+b_{t}$ are linear transformations of t and $x_{i}$ , which respectively represent the target neuron and other neurons in a single channel of the feature map $X\in R^{C\times H\times W}$ . w and b are vectors of weight and bias, whereas M=W×H is the number of neurons in the designated channel. When $\hat{t}=y_{t}$ and ${\hat{x}}_{i}=y_{o}$ , the energy $e_{t}$ attains the minimal value. $y_{t}$ and $y_{o}$ are selected as two different values to find the linear separability between the target neuron *t* and all other neurons in the same channel. For simplicity, binary labels (i.e., $y_{o}=-1$ and $y_{t}=1$ ) are used for them separately, and an additional regularization term $\delta w_{t}^{2}$ is applied to Equation (4). Then, the energy function becomes^46^:

(5) $$e_{t}\left(w_{t},b_{t},y,x_{i}\right)=\frac{1}{M-1}\sum_{i=1}^{M-1}{\left(-1-\left(w_{t}x_{i}+b_{t}\right)\right)}^{2}+{\left(1-\left(w_{t}t+b_{t}\right)\right)}^{2}+\delta w_{t}^{2}$$

When taking the minimum value of Equation (5) by letting $\frac{\partial e_{t}}{\partial w_{t}}=0$ and $\frac{\partial e_{t}}{\partial b_{t}}=0$ , fast closed-form solutions can be derived and given by:

(6) $$w_{t}=-\frac{2\left(t-\mu_{t}\right)}{{\left(t-\mu_{t}\right)}^{2}+2\sigma_{t}^{2}+2\delta }$$

(7) $$b_{t}=-\frac{1}{2}\left(t+\mu_{t}\right)w_{t}$$

where $\mu_{t}=\frac{1}{M-1}\sum_{i=1}^{M-1}x_{i}$ and $\sigma_{t}^{2}=\frac{1}{M-1}\sum_{i=1}^{M-1}{\left(x_{i}-\mu_{t}\right)}^{2}$ are mean and variance calculated over all the neurons except *t* in the designated channel. Since it is reasonable to assume that all pixels in a single channel follow the same distribution, mean and variance can be calculated over all neurons and reused to avoid redundant calculation. Then, the minimal energy can be computed by:

(8) $$e_{t}^{*}=\frac{4\left({\hat{\sigma }}^{2}+\delta \right)}{{\left(t-\hat{\mu }\right)}^{2}+2{\hat{\sigma }}^{2}+2\delta }$$

where $\hat{\mu }=\frac{1}{M}\sum_{i=1}^{M}x_{i}$ and ${\hat{\sigma }}^{2}=\frac{1}{M}\sum_{i=1}^{M}{\left(x_{i}-\hat{\mu }\right)}^{2}$ . Equation (8) indicates the smaller the energy $e_{t}^{*}$ , the more distinctive and important the neuron *t* is. Therefore, the importance of the target neuron can be determined by 1/ $e_{t}^{*}$ . When the energy function is applied to all the neurons and grouped by E . The whole process can be expressed as:

(9) $$\tilde{X}=\mathrm{sigmoid}\left(\frac{1}{E}\right)⊙X$$

Since the sigmoid is a monotonic function and it restricts too large values in E , the relative importance of each neuron remains unchanged.

#### Wise intersection over union

The localization accuracy of the proposed model is determined by the coordinate regression loss $L_{\mathrm{coord}}$ as shown in Equation (3). $L_{\mathrm{coord}}$ is calculated by Intersection over Union (IoU) in many classic object detectors, but IoU may lead to vanishing gradients when applied to deep networks. In this regard, developments such as Generalized IoU,^48^ Complete IoU (CIoU), and Distance IoU^49^ have emerged. CIoU, which incorporates the bounding box overlap with additional terms for center distance and aspect ratio, is deployed in the original YOLOv7 and has been successfully applied to many optical image datasets.^38^ However, distance and aspect ratio in CIoU can aggravate the penalty for low-quality samples, potentially decreasing the generalization performance of the model. Compared with optical images, sonar image datasets are more difficult to label and contain more low-quality samples. Therefore, it is imperative to use a loss function that weakens the penalty of geometric factors when the anchors fit well with the target box.

In the proposed framework, the Wise IoU (WIoU),^50^ which applies a dynamic non-monotonic focusing mechanism, is employed to solve the problem of unbalanced penalty for low-quality examples. Assuming the predicted anchor box is $\vec{B}=\left[x,y,w,h\right]$ and the ground-truth box is ${\vec{B}}_{\mathrm{gt}}=\left[x_{\mathrm{gt}},y_{\mathrm{gt}},w_{\mathrm{gt}},h_{\mathrm{gt}}\right]$ , the IoU loss can be expressed as $L_{\mathrm{IoU}}=1-\mathrm{IoU}$ . WIoUv1 is defined as

(10) $$L_{\mathrm{WIoUv}1}=R_{\mathrm{WIoU}}L_{\mathrm{IoU}}=\exp\left(\frac{{\left(x-x_{\mathrm{gt}}\right)}^{2}+{\left(y-y_{\mathrm{gt}}\right)}^{2}}{{\left(W_{g}^{2}+H_{g}^{2}\right)}^{*}}\right)L_{\mathrm{IoU}}$$

where $W_{g}$ and $H_{g}$ are the dimensions of the smallest enclosing box and * indicates that $W_{g}$ and $H_{g}$ are detached from the computational graph in a specific training epoch. As WIoU does not require an aspect ratio, it is more computationally efficient than CIoU. Furthermore, to reduce the contribution of easy examples to the loss value, a monotonic focusing factor $r={\left(\frac{L_{\mathrm{IoU}}^{*}}{L_{\mathrm{IoU}}}\right)}^{\gamma }$ is introduced by referring to Lin et al.^51^ Then, $L_{\mathrm{WIoUv}2}$ is obtained.

(11) $$L_{\mathrm{WIoUv}2}={\left(\frac{L_{\mathrm{IoU}}^{*}}{L_{\mathrm{IoU}}}\right)}^{\gamma }L_{\mathrm{WIoUv}1},\gamma >0$$

where ${\bar{L}}_{\mathrm{IoU}}^{*}$ is the IoU loss detached from the computational graph, and ${\bar{L}}_{\mathrm{IoU}}$ is a weighted loss, defined as:

(12) $${\bar{L}}_{\mathrm{IoU},t}=\left(1-m\right){\bar{L}}_{\mathrm{IoU},t-1}+m{\bar{L}}_{\mathrm{IoU}}^{*}$$

In this study, $m=1-\sqrt[{\mathrm{pq}}]{0.05}$ , where *p* and q are training epoch and batch size, respectively. The ${\bar{L}}_{\mathrm{IoU}}$ is initialized to 1 to prevent low-quality anchors from being ignored at the early stages of training, that is, making the anchors with higher $L_{\mathrm{IoU}}$ having larger focusing factors. ${\bar{L}}_{\mathrm{IoU}}$ also plays as a normalizing factor to prevent focusing factor *r* from decreasing at the late stages of training, leading to a slow convergence rate. Since the low-quality samples in different datasets have various distributions, the focusing factor can be revised to be non-monotonic and applied to WIoUv1:

(13) $$L_{\mathrm{WIoUv}3}=rL_{\mathrm{WIoUv}1},r=\frac{\beta }{\eta \alpha^{\beta -\eta }}$$

where η makes r=1 when β=η . β is defined as $\beta =\frac{L_{\mathrm{IoU}}^{*}}{L_{\mathrm{IoU}}}$ . The selection of parameters α and η is related to the datasets, which are set as 1.9 and 3.0, respectively, in this article. It can be observed from Equation (13) that WIoUv3 assigns a small focusing factor to low-quality anchors at the middle and late stages of training to reduce the adverse influence of large gradients. In addition, it focuses on normal-quality anchors to improve the localization performance. The WIoUv3 is used as a coordinate regression loss in the proposed framework.

**Figure 3.**
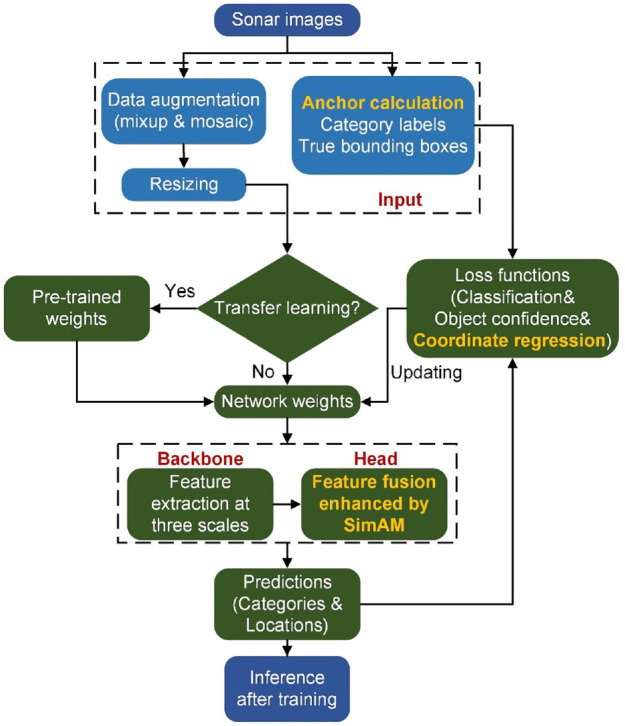
Flowchart of the deployment of the proposed framework.

Overall, three improvements from aspects of anchor initialization, feature fusion, and loss functions are introduced to overcome the limitations of the original YOLOv7. The improvements are marked in bold yellow fonts in Figure 3, which presents the complete process of deploying the proposed framework on sonar images.

## Model evaluation on the public dataset

This section presents a comparative study between the proposed framework and the existing object detection models on the UATD dataset,^36^ an open-access dataset for UOD using multibeam forward-looking sonar (MFLS).

### Overview of the UATD dataset

**Figure 4.**
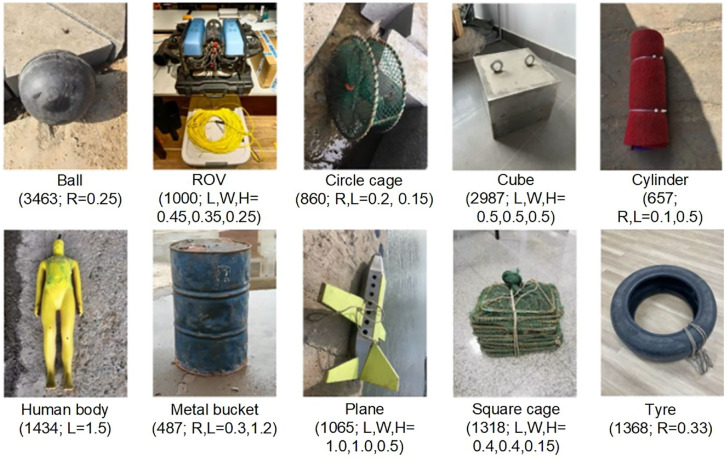
Objects and their sample numbers in the UATD dataset. UATD: underwater acoustic target detection.

Underwater data collection with sonar devices often comes with a high cost and requires professional experience; thus, there are very few publicly available datasets for UOD applications. UATD is one of the few public datasets suitable for object detection of underwater structures. This dataset was collected using Tritech Gemini 1200ik MFLS in lake water with a depth of 4–10 m, and it contains 9200 images in BMP format and their corresponding annotation files in XML format.^36^ The dataset is divided into three archives, including 7600 pairs of data for training and 800 pairs of data as two testing sets (namely “UATD_Test_1.zip” and “UATD_Test_2.zip”). Annotation files include the information of object category names and bounding box coordinates. A total of 10 object categories of sonar images were collected with two different frequencies (720 and 1200 kHz), while the sonar working range was kept between 5 and 25 m. Figure 4 shows each object with its dimensions (in m) and the number of samples included in the UATD dataset. L, W, H, and R represent length, width, height, and radius, respectively.

**Figure 5.**
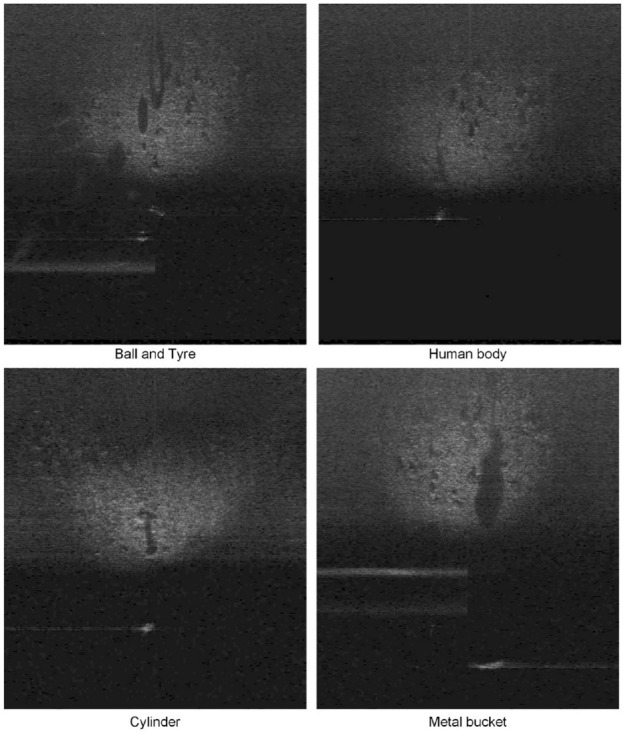
Sonar imaging of some objects selected from the UATD dataset. UATD: underwater acoustic target detection.

It is noteworthy that the dataset contains the basic shapes that make up engineering structures, such as cubes and cylinders; thus, it can be used as pre-training data for detection models of underwater structures, for example, bridge piers and abutments. To provide a more intuitive illustration for the UATD dataset, Figure 5 presents sonar images of basic shapes of objects that may be encountered in underwater structural inspections. The presented images have been processed through gamma correction with *γ* = 2.5^52^ to improve the contrast between the foreground and background. The original images used for training and evaluation are nearly black, and the objects are not clearly visible. It highlights the increased difficulty of object detection based on sonar images compared to conventional object detection tasks.

### Comparative analysis

To evaluate the performance of the proposed model, Faster-RCNN, YOLOv3, and YOLOv7 are selected for comparative analysis. Faster-RCNN and YOLOv3 are renowned as the most popular two-stage and one-stage object detectors, respectively. In the analysis, they are integrated with various backbones. In addition, YOLOv7, a state-of-the-art detector not previously applied to UOD, is employed to ascertain the efficacy of the three improvements introduced in the proposed framework.

The performance evaluation compares both the accuracy and efficiency of the three models. To measure the accuracy of detectors on UATD, two primary metrics are adopted: mean average precision at an IoU threshold of 0.5 across all categories (mAP_0.5_) and mean average recall (mAR). mAP indicates the ability of a model to accurately identify positive examples and to minimize false positives, while mAR reflects the ability to find out all the positive examples. On the other hand, the efficiency is evaluated by the parameter size of models, denoted as “Params.” All the detectors are implemented using PyTorch and are trained on the training set of UATD on a local computer equipped with an NVIDIA RTX A6000 GPU. Input sonar images are uniformly resized into the resolution 512 × 512 before training, and models are initialized with pre-trained weights from COCO.^53^ For consistency across different model evaluations, training parameters are set uniformly. Each model undergoes training for 200 epochs with early stopping criteria. An initial learning rate of 0.0005 is set, with adaptive adjustments using a cosine annealing schedule. A batch size of eight is chosen, and the Adam optimizer is employed for all models.

The first testing set (i.e., “UATD_Test_1.zip”) is selected to evaluate the trained models. The evaluation results are listed in Table 1, where the best results are shown in bold font. It can be observed from Table 1 that the proposed model achieves the best mAP_0.5_ and mAR, with a moderate scale of parameters. Compared with the official version of YOLOv7, the proposed model keeps the parameter scale unchanged but achieves significant performance improvements in almost all categories, with only two categories, cube and cylinder, showing a slight decrease in performance. Compared with the other two improvements, the SimAM module contributes mostly to the model performance. In addition, it is noticed that the optimization of the model structure has different effects on various objects. For example, when using a low-parameter backbone network in Faster-RCNN and YOLOv3, objects of the circle cage and square cage achieve the best performance, respectively. Furthermore, it is necessary to note that improvements in model performance are not always accompanied by increases in model parameters. These findings are not common in object detection for optical images. The reasons may include the following points:

1. The quality of optical imaging is independent of the type of object, while the quality of sonar imaging is closely related to the shape, size, and material of the object being detected. It may be that the materials of the circle cage and square cage cause diffuse reflection of sound waves, resulting in sonar image features that are not significant enough.
2. Sonar images are typically grayscale and have lower resolution and therefore, have fewer image features. While the training data remains unchanged, excessively increasing the complexity of the detector may lead to overfitting and failure to improve detection results.

**Table 1.** Object detection results on UATD.

Model	AP_ball_	AP_sc_	AP_cube_	AP_plane_	AP_tyre_	AP_cc_	AP_hb_	AP_cy_	AP_mb_	AP_rov_	mAP	mAR	Params
Faster-RCNN + ResNet-18	74.5	**70.4**	65.7	94.4	87	71.2	79	97.3	100	93.9	83.3	77.6	28.17M
Faster-RCNN + ResNet-50	74	69.3	57.7	93.8	88.8	68.7	79.6	95.5	100	93.4	82.1	74.3	41.17M
Faster-RCNN + ResNet-101	73.5	63.3	59.6	96.5	89.6	62.9	80.5	99.2	100	96.2	82.1	76.7	60.16M
YOLOv3 + Darknet53	81.2	57.1	67.2	93.2	76.9	72.2	69.5	96.1	96.7	100	81	56.6	61.57M
YOLOv3 + MobileNetV2	79.7	59.6	59.5	97.5	84	**75.8**	82.2	95.2	98.5	98.5	83.1	73.5	3.68M
YOLOv7	81.8	66	74.6	98.6	93.3	52.4	97.3	99.2	100	99.9	86.3	88.4	37.67M
YOLOv7 + *k*-means*++*	80.9	64.0	75.0	99.1	94.2	60.4	97.0	99.5	100	100	87.0	88.8	37.67M
YOLOv7 + WIoU	82.2	65.0	76.3	98.8	93.7	60.4	97.3	**99.5**	100	99.7	87.3	88.6	37.67M
YOLOv7 + SimAM	82.6	66.5	**81.5**	**99.1**	92.4	59.3	97.6	98.7	100	100	87.8	88.6	37.67M
Proposed method	**86.1**	67.1	74.4	99	**96.9**	60.3	**100**	98.4	**100**	**100**	**88.2**	**89.1**	37.67M

AP_sc_, AP in square cage; AP_cc_, AP in circle cage; AP_hb_, AP in the human body; AP_cy_, AP in cylinder; AP_mb_, AP in a metal bucket; AP: average precision; mAP: mean average precision; mAR: mean average recall; UATD: underwater acoustic target detection. The unit of AP, mAP, and mAR is %.

Even though sonar imaging quality varies from object to object, the proposed UOD model shows clear advantages in most categories, as shown in Table 1. The above analysis also reveals that sonar-based UOD can be significantly affected by the characteristics of detected targets, which is more complex than optical object detection; therefore, the selection of detectors should be based on specific application. Since the available sonar datasets are very limited and collecting sonar data are costly, testing new methods on existing datasets and applying them to similar targets in new scenarios through transfer learning is significant.

## Experimental study

To investigate the feasibility of the proposed framework for transfer learning, this section presents an independent experimental study using an ROV equipped with MFLS. The following subsections provide details of the experiment and a discussion of the results when applying the proposed approach to UOD.

### Experimental setup

**Figure 6.**
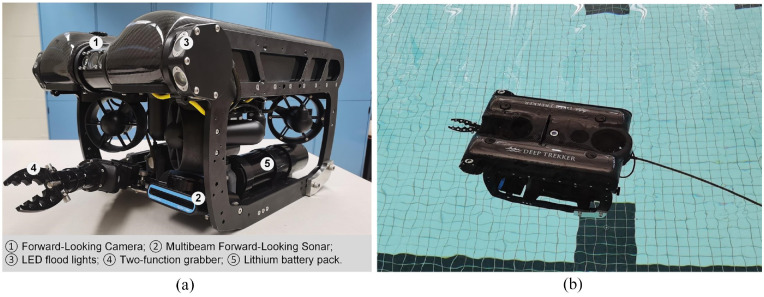
The sonar-equipped remotely operated vehicle: (a) components and (b) operation in the water.

In the experiment, an ROV from Deep Trekker called Pivot (manufacturer: Emesent), is employed as the platform for data collection. As shown in Figure 6, the ROV has a built-in optical camera, multibeam sonar, LED flood lights, and a two-function grabber. The device has a depth rating of 305 m. While in operation, the device is connected to the controller using a tether cable, which transmits commands from the controller, and sensor readings from the ROV. The tether supports up to 136 kg and can be used to reel the ROV back to the surface. The ROV is propelled using six electric thrusters, weighs approximately 17 kg, and its dimensions are 360, 310, and 576 mm. Besides the optical cameras, the other main sensory device on this ROV is an Oculus M-series multibeam sonar. This sonar device allows the ROV to navigate and collect data in low visibility conditions. The technical parameters of the sonar are given in Table 2. To achieve higher resolution in sonar images, high-frequency mode (i.e., 3 MHz) was applied to detect objects in the range of 0.1–5 m. The camera in this study is only used for remote control assistance, whereas the sonar is used to collect imaging data of objects.

**Table 2.** Technical parameters of the sonar.

Model	Frequencies	Range	Range resolution	Horizontal aperture	Vertical aperture	Number of beams	Angular resolution
Oculus M3000d	1.2 MHz/3 MHz	0.1 m–30 m/5 m	2.5 mm/2 mm	130°/40°	20°/20°	512	0.6°/0.4°

**Figure 7.**
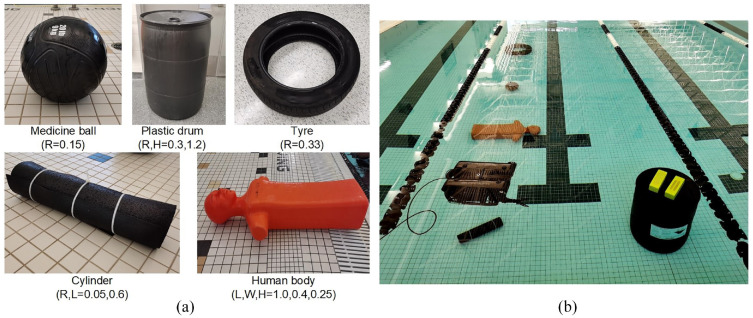
Items for object detection: (a) individual photos and (b) arrangement in the water.

The experiment is conducted in an aquatic center with a water depth of 1.5 m and a water temperature of 27°C. To facilitate capturing objects from various angles, the diving depth of the ROV is controlled at 0.4 m. Since underwater engineering structures are primarily composed of simple geometric shapes, such as cylinders, rectangular prisms, and the like, this experiment selects five highly relevant object classes from the UATD dataset for transfer learning analysis. Figure 7 presents the five items with a similar appearance to the five categories of the ball, metal bucket, cylinder, human body, and tire in UATD. The objective of transfer learning is to utilize the weights of detection models trained on the publicly available UATD dataset to assist in the detection of similar objects during new experiments. The item dimensions (in m) are shown to demonstrate the detection ability for objects of different scales.

**Figure 8.**
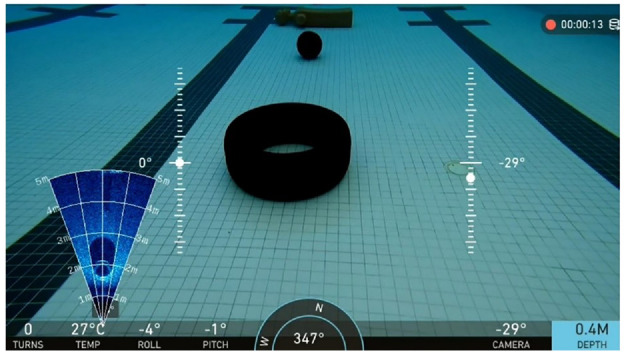
Visual data including image and sonar data recorded by the ROV system. ROV: remotely operated vehicle.

The camera and sonar mounted on the ROV are automatically synchronized, recording visual data in video format, and an example of the recorded data is shown in Figure 8.

### Data processing

**Figure 9.**
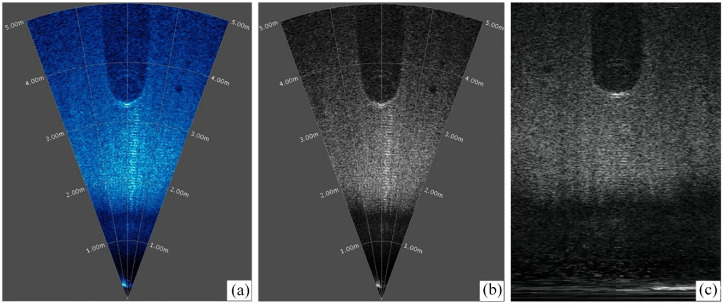
Processing of sonar images: (a) raw data, (b) grayscale, and (c) conversion to rectangle.

The raw data obtained from the sonar is initially processed by the accompanying software Oculus Viewpoint, yielding a default representation in the form of a blue sector pattern. As illustrated in Figure 9(a), the sector pattern is a frame extracted from the recorded videos and corresponds to the imaging output of a tire. The angular extent of the sector represents the horizontal viewing angle of the sonar, while the radius length corresponds to the detection distance. Therefore, the object dimensions and their relative positions concerning the ROV can be determined accordingly. To enhance the discriminability between foreground and background within the images, the raw images are first grayscale processed, as shown in Figure 9(b). Subsequently, a polar coordinate transformation is applied to convert the sector-based representation into a rectangular image, as depicted in Figure 9(c). It should be noted that the objects may exhibit some distortion in the transformed images, but this does not significantly impact the detection of their positions. Since the training and inference of the UOD model are performed on distorted images, accurate rectangular bounding boxes in these images ensure that, when inversely transformed, compact bounding boxes shaped as curved polygons can also be obtained, identifying targets in the original sonar imagery.

In the experiment, the ROV is employed to approach the target objects from both directly above and from their lateral sides, allowing for the acquisition of sonar images with both top-down and side-view. A total of 1500 rectangular images, each with a resolution of 673 × 965 pixels, were randomly selected and processed for five categories of objects. Then, manual annotations are conducted to specify the object categories and their respective positions within the images, that is, the ground-truth bounding box ${\vec{B}}_{\mathrm{gt}}=\left[x_{\mathrm{gt}},y_{\mathrm{gt}},w_{\mathrm{gt}},h_{\mathrm{gt}}\right]$ .

It is worth noting that the deliberate use of a relatively small dataset is intended to assess the transfer learning capabilities of the proposed UOD framework under limited data conditions, which holds significant relevance for practical applications. The dataset is divided into training and testing sets in a ratio of 9:1 and then used for the training and evaluation with the same procedure introduced in the previous two sections.

### Results and discussions

To perform a comparative analysis of model transferability, three different training strategies are employed for both the proposed framework and the original YOLOv7, including training without pre-trained weights, transfer learning with weights trained on the COCO (the abbreviation of common objects in context), and transfer learning with the weights trained on the UATD dataset. The hyperparameter settings of training remain consistent with those outlined in Comparative analysis.

**Figure 10.**
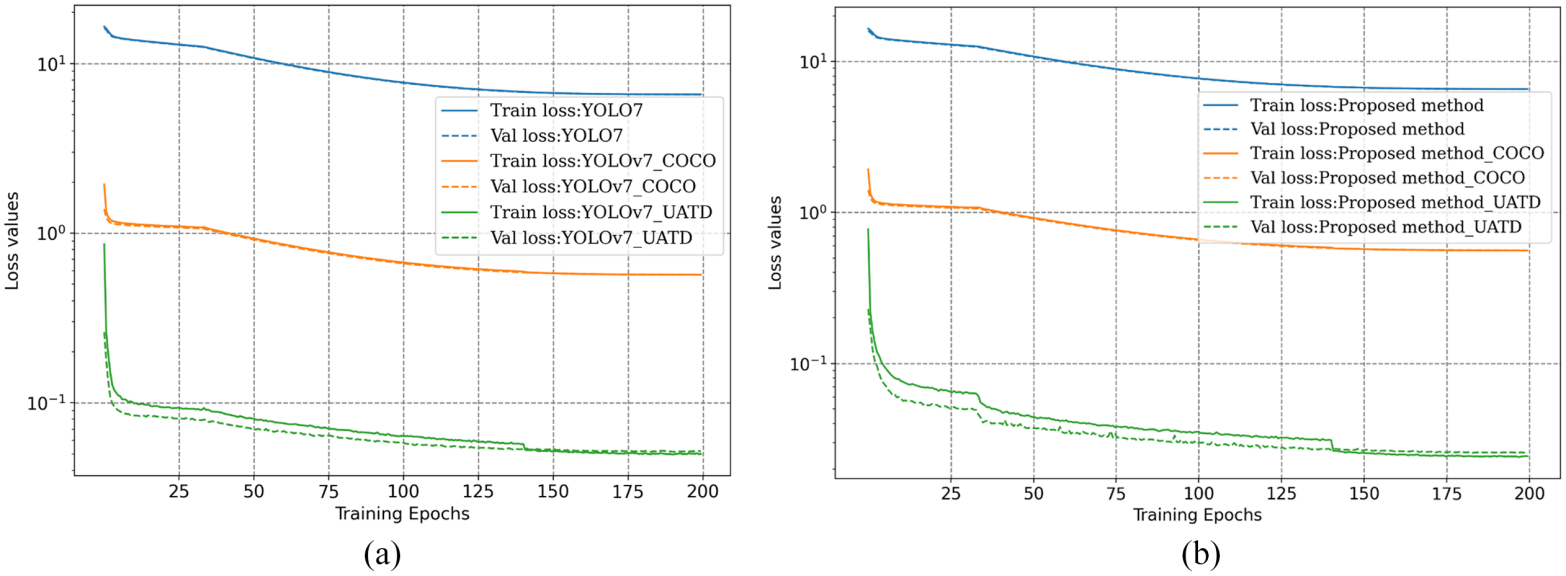
Training and validation loss curves of the transfer learning with different initial weights: (a) YOLOv7 and (b) the proposed method.

In the training of the proposed framework and YOLOv7, only the initial weights are changed in the models. Therefore, for the same model, the value of loss functions during the training process can provide an initial assessment of the model performance. Figure10 shows the curves of training and validation loss for both models under three different initial training weights. It is shown that both the training and validation losses converge steadily to a stable stage in all training situations, indicating the absence of significant overfitting. However, from the perspective of loss values, the models trained without pre-trained weights are presented with the poorest performance, whereas the models trained with the UATD weights can outperform those with the COCO dataset weights. It illustrates that even though the measured data significantly differs from the UATD datasets, as can be observed by comparing Figures 5 and 9, applying transfer learning and the pre-trained weights from related datasets are beneficial and necessary for UOD applications in unknown scenarios.

**Figure 11.**
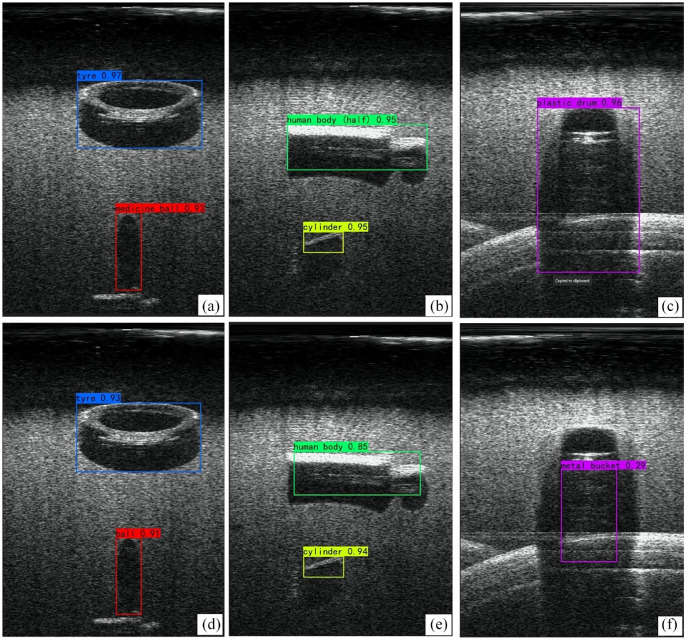
Detection results of similar objects using the transfer learning from UATD initial weights: (a–c) the proposed approach and (d–f) the original YOLOv7. UATD: underwater acoustic target detection.

Figure 11 presents the object detection results in the testing set of the proposed framework and YOLOv7, which were trained from the UATD initial weights. It is evident that despite the relatively low resolution of sonar images and limited foreground–background contrast, all objects in the images are correctly classified by both the YOLOv7 and the proposed approach. However, it can be observed that the proposed approach yields higher classification probability scores. More importantly, the bounding boxes predicted by the proposed approach more closely fit the object contour, indicating a higher level of precision in localization. In addition, the detection outcomes remain unaffected by shadows, as can be seen in Figure 11(b). These results demonstrate that the proposed UOD model can successfully detect targets as small as 5 cm (i.e., cylinder) through transfer learning. This approach shows promise for application in underwater SHM, particularly in detecting apparent damages such as spalling and scouring. Since sonar images contain depth and size information, when coupled with the segmentation of pixels, a further quantitative assessment of apparent damages can also be achieved. However, it is important to note that relying solely on the sonar for the detection of small damages, such as cracks, is challenging because sonar images inherently have low resolution and increased background noise. The ROV used in this study is equipped with an optical camera, and a simple way to boost small damage assessment is to fuse information from the camera and sonar.

**Figure 12.**
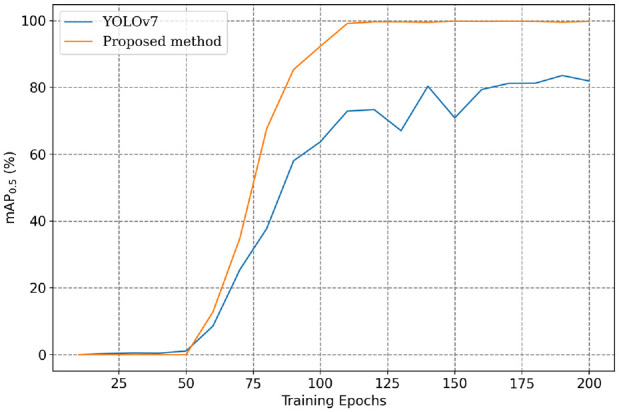
Comparison of mAP curves when training without pre-trained weights. mAP: mean average precision.

In addition to assessing the superiority of the proposed approach over YOLOv7 in object classification, further analysis was conducted to compare the mAP of the two models at different training stages. On one hand, concerning the scenario of training from scratch, Figure 12 presents the mAP curves for the entire 200 training epochs. Both models exhibit small variation in mAP during the first 50 epochs. This is because the backbone of the models was frozen in the initial 50 epochs to reduce memory consumption, and all parameters of the model were updated beyond the 50th epoch. Figure 12 clearly shows that under the condition of a limited training dataset, the proposed approach demonstrates a faster and more stable performance improvement. Upon completion of training, the proposed approach achieved an mAP close to 100%, whereas YOLOv7 reached approximately 80%.

**Figure 13.**
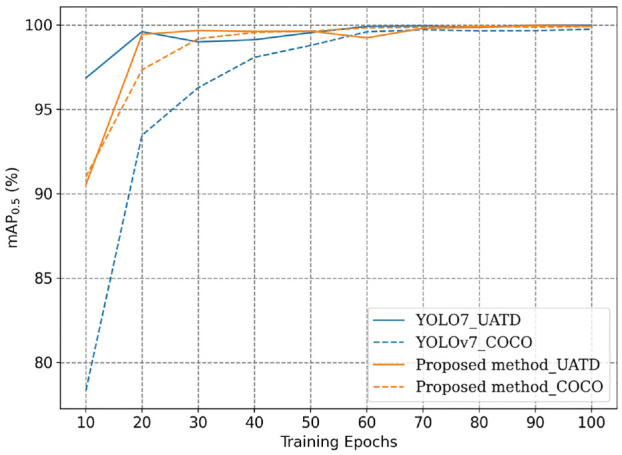
Comparison of mAP curves when training from different initial weights. mAP: mean average precision.

On the other hand, to assess the transferability of the detection models, the weights from both the COCO and UATD datasets were used for model training. Figure 13 shows the mAP curves of the two models with different initial weights. From the observations in the figures, the following findings can be discerned:

1. When the weights of the general dataset COCO are used for transfer learning, the training speed of the proposed framework is significantly faster than YOLOv7. The two models mostly reach optimal performance at around 60th epoch, though a slight advantage in mAP is still retained by the proposed approach.
2. When training with UATD weights, both models swiftly attain optimal performance, and the mAP performance is notably superior compared to using COCO weights. This outcome was expected since the data characteristics of UATD more closely align with the measured data in this experiment. Additionally, it underscores the necessity of establishing specialized datasets for UOD tasks.

The experimental results conclusively demonstrate that the proposed framework outperforms YOLOv7 in both object classification and localization tasks while showcasing superior transfer learning capabilities. However, it should be mentioned that this study primarily focuses on methodological research for UOD, and the experimental data utilized were obtained under controlled conditions (i.e., an aquatic center). When applying the framework to actual underwater infrastructure inspection using sonar and ROV, its performance is influenced by more environmental factors such as water depth, turbidity, temperature, etc. Therefore, further field experiments and case studies on underwater structures will be pursued for future work.

## Conclusions

For sonar-based inspection of underwater infrastructure, this article proposes a novel object detection framework by employing three improvements in the state-of-the-art method YOLOv7. Systematic comparative studies and underwater ROV experiments demonstrate that this framework is superior to existing mainstream approaches. Based on the results obtained, the following conclusions can be drawn.

1. The UOD framework presented in this article adopts the basic architecture of YOLOv7 and introduces improvements in three dimensions: anchor initialization, adaptative feature fusion, and effective loss functions. Test results indicated that all three improvements significantly boost model performance while reducing the model dependency on the scale of datasets. Adaptive feature fusion contributes the most to improving model performance.
2. To the best of the authors’ knowledge, YOLOv7, as one of the state-of-the-art visual models, has not been previously reported in the context of underwater object inspection using sonar. Through a comparative analysis of the publicly available UOD dataset (UATD), the proposed framework was demonstrated to achieve the highest mAP among existing approaches, all while maintaining a comparable level of complexity.
3. The transferability of the proposed framework was validated through indoor experiments with an underwater ROV. The results successfully demonstrated that the suggested model exhibits faster learning speed and better outcomes on unfamiliar datasets compared to the original YOLOv7, showing strong potential for cross-dataset transfer learning.

This research explores a novel deep learning-based UOD framework for underwater infrastructure inspection and monitoring and presents a preliminary application of a sonar-equipped ROV. In future studies, the effectiveness of the proposed framework in real-world underwater structural inspection and its potential integration with ROVs remain to be explored.
